# Optical observations of thunderstorms from the International Space Station: recent results and perspectives

**DOI:** 10.1038/s41526-023-00257-4

**Published:** 2023-02-04

**Authors:** Torsten Neubert, Francisco J. Gordillo-Vázquez, Heidi Huntrieser

**Affiliations:** 1 National Space Institute, Technical University of Denmark (DTU Space), Elektrovej bld. 328, 2800 Kongens Lyngby, Denmark; 2 Instituto de Astrofísica de Andalucía (IAA - CSIC), Glorieta de la Astronomía s/n, 18008 Granada, Spain; 3 Institute of Atmospheric Physics, German Aerospace Center, Münchner Straße 20, 82234 Oberpfaffenhofen-Wessling, Germany

**Keywords:** Environmental sciences, Plasma physics, Environmental chemistry

## Abstract

The International Space Station (ISS) is in the lowest available orbit at ~400 km altitude, bringing instruments as close to the atmosphere as possible from the vantage point of space. The orbit inclination is 51.6°, which brings the ISS over all the low- and mid-latitude regions of the Earth and at all local times. It is an ideal platform to observe deep convection and electrification of thunderstorms, taken advantage of by the Lightning Imaging Sensor (LIS) and the Atmosphere Space Interaction Monitor (ASIM) experiments. In the coming years, meteorological satellites in geostationary orbit (~36,000 km altitude) will provide sophisticated cloud and lightning observations with almost complete coverage of the Earth’s thunderstorm regions. In addition, Earth-observing satellite instruments in geostationary- and low-Earth orbit (LEO) will measure more atmospheric parameters at a higher resolution than we know today. The new infrastructure in space offers an opportunity to advance our understanding of the role of thunderstorms in atmospheric dynamics and climate change. Here, we discuss how observations from the ISS or other LEO platforms with instruments that view the atmosphere at slanted angles can complement the measurements from primarily nadir-oriented instruments of present and planned missions. We suggest that the slanted viewing geometry from LEO may resolve the altitude of electrical activity and the cloud structure where they occur, with implications for modelling thunderstorms’ effects on the atmosphere’s radiative properties and climate balance.

## Introduction

**Fig. 1:**
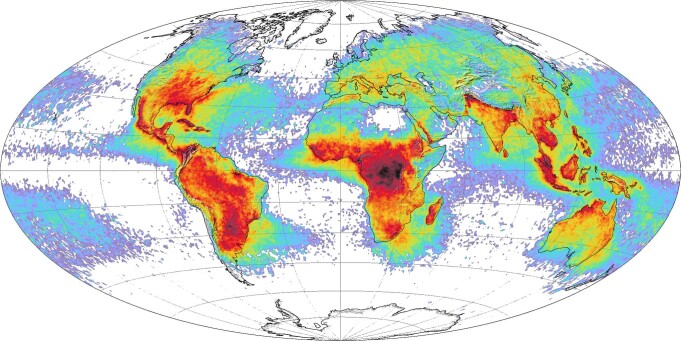
Global annual lightning flash density rates. The colours mark flashes/km^2^/year from 0,1 (magenta) to 70 (black). The data are from the OTD and TRMM-LIS (1995-2003). Based on a NASA image by Marit Jentoft-Nilsen, with data provided by the Global Hydrology and Climate Center Lightning Team^102^.

Thunderstorms develop primarily at low- and mid-latitudes, where the solar energy input is the largest. The atmosphere over land is heated unequally depending on the underlying surface, and thermal bubbles develop^1^. Some may rise as deep convection to the upper tropopause, occasionally even into the lower stratosphere^2^. Deep convection carries high amounts of water vapour, dust, aerosols, and trace gases from the polluted boundary layer that may reside at high altitudes for times much longer than the duration of the storm (days versus hours). Spreading over extended regions (~100–1000 km), they perturb the radiative properties of the upper troposphere and lower stratosphere (UTLS) region^3–5^. Lightning affects trace species’ concentrations by specific chemical reactions in the heated lightning channel^6–8^. It can cause deaths and injuries, crop and property damage, and may ignite wildfires^9^ that release huge quantities of trace species (including greenhouse gases) into the atmosphere. Locally and temporally these amounts might exceed anthropogenic emission of such gases^10^. The emissions may be injected into the stratosphere^11,12^ and are important contributors to global warming and climate change^13^. Concurrently, climate change increases the frequency of hot and dry weather situations that fuel wildfires. Studies on lightning activity in a warmer climate suggest that the average global activity may decrease because of a diminishing amount of hail in thunderstorms^14^, whereas regional activities may increase^15,16^. Especially in the high Arctic region, where wildfires are easily induced, a drastic rise in lightning activity has been observed^17^, causing a rapid release of trace species with limited possibilities to quench the fires. For these reasons, the World Meteorological Organisation included lightning as an essential climate variable^18^. It is of interest, then, to understand how thunderstorms and lightning activity, on the one hand, affect the climate balance and, on the other hand, how they are affected by a changing climate. Here, the ISS is in a well-suited orbit, passing over all the major thunderstorm regions of the Earth within ±51.6° latitude, as shown in Fig. 1.

In the following, we give a brief account of the development of optical observations of lightning activity from space. We discuss some aspects of how thunderstorms may affect the radiative properties of the atmosphere and point out that the altitude of perturbations to atmosphere of greenhouse gases is important. We end by discussing the opportunities for using the ISS, or another platform in LEO, for future observations with slanted viewing geometry that allows for measurements of thunderstorm activity with resolution in altitude, complementing the primarily nadir-viewing measurements of current and planned missions.

## Observations

### The first instruments for optical lightning detection from space

Optical observations of lightning from low-earth orbit were first conducted by the Optical Transient Detector (OTD) on the MicroLab-1 satellite launched in 1995 into a 70° orbit at 740 km altitude^19^. The instrument represented a technological breakthrough because it could measure lightning flashes during the day in a background of light reflected from solar-illuminated clouds. This capability was achieved by selecting a narrow spectral band (~1 nm) around a strong line of the lightning spectrum (an atomic oxygen line at 777.4 nm), a pixel resolution that matches the cloud illumination by a flash (~10 km^2^), and a time resolution that fits the typical duration of optical pulses (2 ms). Excited atomic oxygen is almost absent in the troposphere except in the high-temperature channels of lightning leaders, and is a unique signature of a lightning flash. The OTD gave the first global map of lightning climatology by a single sensor with good statistics of all the thunderstorm regions of the Earth at all local times and seasons^19^.

The Lightning Imaging Sensor (LIS), based on the OTD, was included in the payload of the Tropical Rainfall Measuring Mission (TRMM) satellite. TRMM was launched in 1997 into an orbit at 350 km altitude and 35° inclination. The orbit was raised to 402 km in 2001 and re-entered the atmosphere in 2015^20,21^. LIS data were used to detect deep convection without land-ocean bias, estimate the precipitation mass in the mixed-phase region of thunderclouds, and differentiate storms with strong updrafts from those with weak vertical motion.

The OTD and LIS data provided a wealth of information on the climatology of lightning day and night, such as lightning flash rate variability with location over the globe^22^, hot-spot regions, and variations with local time and season^19^ at geographic scales down to 0.5°^23^ and 0.1°^24^, respectively. A study of lightning activity on a decadal-length time scale showed that the activity measured by LIS remained roughly constant, while a ground-based detection system found a ~13% reduction in the continental United States^25^. Other studies show that when considered on a global scale, the relationship between column-integrated precipitation ice mass and lightning flash density is almost linear and invariant between land, ocean and coastal regimes in contrast to liquid precipitation^26^.

Furthermore, imaging instruments such as OMI on Earth observation satellites can detect NO_X_ (=NO + NO_2_) perturbations by lightning^27,28^, which are produced in high amounts within the hot lightning channel and may impact the ozone budget in the UTLS region.

Ground-based lightning detection systems with global coverage have also been fielded during the past decades. They are based on measurements of pulses of electromagnetic waves in the VLF-LF – range (3–300 kHz) emitted by lightning propagating in the earth-ionosphere wave guide^29–31^. They appear to detect less lightning activity than OTD/LIS, with a more variable cover of the Earth because of the uneven distribution of wave receivers. They may be more sensitive to low-altitude cloud-to-ground lightning, whereas OTD/LIS is relatively more sensitive to intra-cloud lightning in clouds at higher altitudes^32^. Lightning Mapping Arrays (LMAs) have also been installed at select locations. They measure lightning processes in three dimensions (also altitude) over regions of several hundred km radii with a time of arrival technique of pulses^33^.

The OTD and LIS were pathfinders for the first-generation lightning detectors on weather satellites in geostationary orbit. One is the Geostationary Lightning Mapper (GLM) on the U.S. GOES-R and GOES-S satellites (GOES-16 and GOES-17), launched in 2016 and 2018, covering the Americas^34^. China has the Lightning Mapper Imager (LMI) on Fengyun-4A and 4B, launched in 2015 and 2021, covering China and East Asia^35^. Finally, Europe has developed the Lightning Imager (LI), launched on the Meteosat Third Generation (MTG-I) satellite on 13. December 2022, to cover Europe and Africa^36^.

### Observations by instruments on the ISS

The LIS instrument was installed on the ISS in 2017 to continue monitoring lightning activity, providing the longest record of activity from LEO with the same instrument^37,38^. It allowed for coverage of regions within ±70° latitude by OTD, ± 35° by TRMM-LIS, and ±51.6° by ISS-LIS, securing lightning observations that cover more than 25 years, allowing studies of climate–lightning relationships^38^. Some recent publications based on ISS-LIS measurements discuss the cross-correlation of ground-based lightning detection systems to estimate the detection efficiencies of measurement techniques such as the LINET 3D system^39^ and a lightning detection network in China^40^. Other papers study the quantification of NO_*X*_ based on lightning and TROPOMI measurements^41^, parametrization of NO_X_-production in continuing currents^42^, and meteorological conditions for lightning-ignited fires^43^.

Recent studies also compared ISS-LIS observations with those of ASIM, GLM, and an LMA system in Colombia^44,45^. ASIM was launched to the ISS 2. April 2018. The payload includes three photometers at 180–235 nm (UV), 337 nm with 4 nm bandwidth (blue), and 777.4 nm with 5 nm bandwidth (red), and two cameras observe in the same blue (4 nm) and red bands (3 nm). The photometers sample at 100 kHz and the cameras take 12 frames per second at 1 Mpixels/frame and ~400 m resolution at cloud tops. The instruments point towards the nadir and measure only during the night^46^.

ASIM aims to study Transient Luminous Events (TLEs) in the atmosphere above thunderstorm clouds such as the sprites, jets, and gigantic jets^47,48^, the Terrestrial Gamma-ray Flashes (TGFs)^49^ from thunderstorms, and conventional lightning^50^. One of the arguments for ASIM was that TLEs and TGFs, while being fascinating by themselves, also are a window into the inner workings of lightning, where the fast time scales and difficulties with in situ measurements in thunderclouds place severe challenges on the measurements. Thus, although studied since the time of Jacques de Romas and Benjamin Franklin, many aspects are unknown such as how lightning initiates in clouds, how high the electric fields in clouds must be to trigger lightning, and the microphysics of lightning^51^. Therefore, the blue band was selected for ASIM because it is a line of N_2_ primarily emitted by streamers. Streamers are ionisation waves known to be launched from the tips of lightning leaders or pointy conductors in high-voltage potentials, where they form a streamer corona^52^, but they can also occur as isolated discharges in clouds^53^. The red band was chosen because it is a strong signature of the lightning leader and is used by LIS and other optical lightning detection imagers. Each spectral band is then sensitive to the two fundamental modes of atmospheric electrical activity: the hot leader (lightning) and the cold streamer (coronas). These modes also form most of the TLEs in the atmosphere above thunderstorms. They are easier to observe here because the temporal and spatial scales are approximately proportional to the mean free path of electrons, which can be orders of magnitude longer in the stratosphere and mesosphere.

**Fig. 2:**
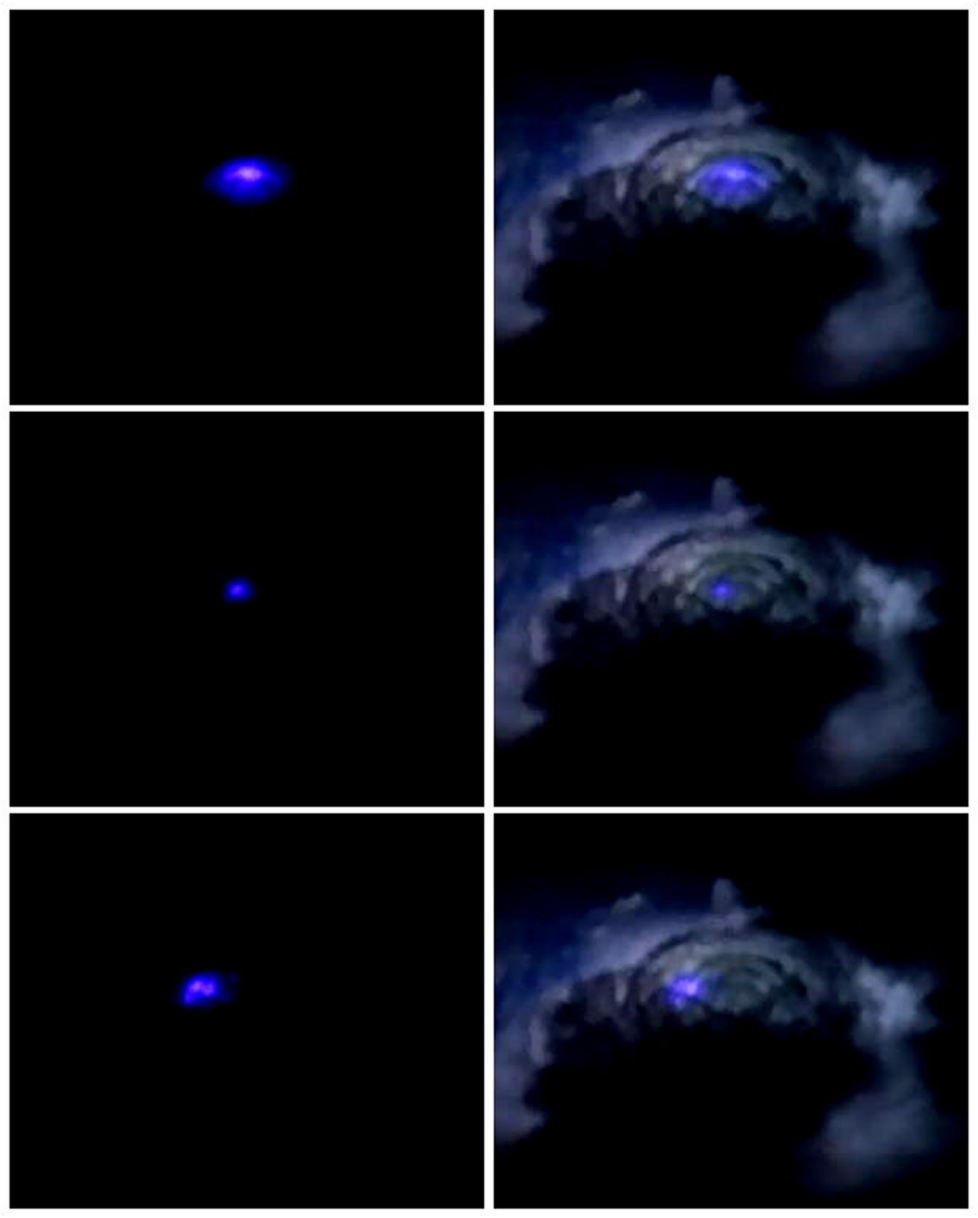
Blue corona discharges at cloud tops observed by the THOR experiment. On the left, the discharges as observed, and on the right, superimposed on the cloud illuminated by internal lightning^54^.

The ASIM experiment was preceded by the THOR experiment of Danish astronaut Andreas Mogensen in 2015. With a NIKON D4 colour video camera, he captured profuse activity in the top layer of a thunderstorm cell on the Indian east coast^54^. Some examples are shown in Fig. 2. The observations underscored that the upper regions of thunderstorm clouds are particularly poorly researched because they are difficult to measure from the ground.

**Fig. 3:**
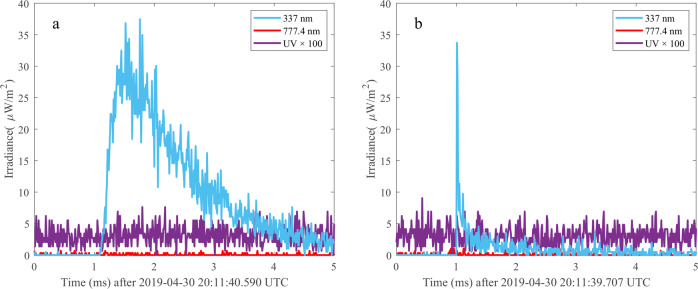
Blue corona discharges measured by the ASIM photometers. **a**) With rise time ~50 μs and **b**) with rise time within one sample of 10 μs.

The ASIM measurements confirmed that blue streamer discharges are common in the upper regions of deep convective cloud cells^55–57^. Figure 3 shows examples of the two most commonly observed discharges. They consist of a single blue flash with relatively faint emissions in the red band, suggesting the emissions are from corona streamers. In Fig. 3a, the rise time of the blue pulse is several samples, and in Fig. 3b, the pulse peaks within 1 sample (10 μs). Assuming the source of the emissions is short compared to the sampling period, the rise times are interpreted to be from the scattering of photons in cloud hydrometeors and then reflect the depth of the discharges in the clouds^52^. The pulse with a fast rise time in Fig. 3b is then from a discharge at the top of a cloud, whereas the one with a longer rise time in Fig. 3a is from a source some kilometres below the cloud top^57^.

Blue corona discharges appear to be the optical equivalent to the so-called Narrow Bipolar Events (NBE) in radio signals from thunderstorms. These are short (10–20 µs) bipolar pulses with very high power in the High Frequency (3–30 MHz) and Very High Frequency (3–300 MHz) bands^58^. They are thought to be fast breakdown and may occur at the initiation of lightning flashes^59^. Correlating the ASIM blue discharges with NBEs observed by ground lightning networks, a consistent picture appears where negative NBEs, which tend to occur at the top of clouds, correlate with fast blue pulses, and positive NBEs, most common within the clouds, with the slower pulses^52,60^. Multi-pulse blue discharges are also observed in relation to NBEs where the subsequent pulses could be caused by leader branching^61^. In addition, ASIM observed a blue corona discharge at the initiation of lightning^62^ and at the onset of a blue jet into the stratosphere^63^, suggesting that corona discharges and NBEs may be integral to the initiation of both lightning and upward lightning in the form blue jets.

The discovery of blue corona discharges in thunderclouds follows that of sprites and blue jets (1990, 1994)^47^, TGFs of radiation reaching several tens of MeV (1994)^49^, and gigantic jets from cloud tops to the ionosphere (2002)^48^. Blue emissions at cloud tops with little apparent leader activity were first observed from the ground and reported in 2003^64^ and 2011^65^. Observations followed by the Imager for Sprites and Upper Atmospheric Lightning (ISUAL) on the FORMOSAT-2 satellite^66,67^. However, the measurements by ASIM, combined with simultaneous measurements by ground-based radio receivers, proved they are from corona streamers^52^.

## Perspectives on lightning observations from the ISS

In the following, we first discuss the merits of continued observations of thunderstorms from the ISS in a slanted viewing geometry that allows for altitude resolution of discharges and cloud structure. Our main point is that the change to the atmosphere’s radiative properties depends on the altitude of the perturbations^68,69^. It is important, therefore, to measure the altitude of electrical activity and cloud structure to understand the role of thunderstorms in a changing climate. From the ISS, we can get such measurements at high spatial resolution. The discussion is also valid for other LEO space platforms of the future. We end with comments on the synergy of current and planned Earth observation missions and the new lightning mappers on geostationary satellites.

### Perturbations to greenhouse concentrations by corona discharges

**Fig. 4:**
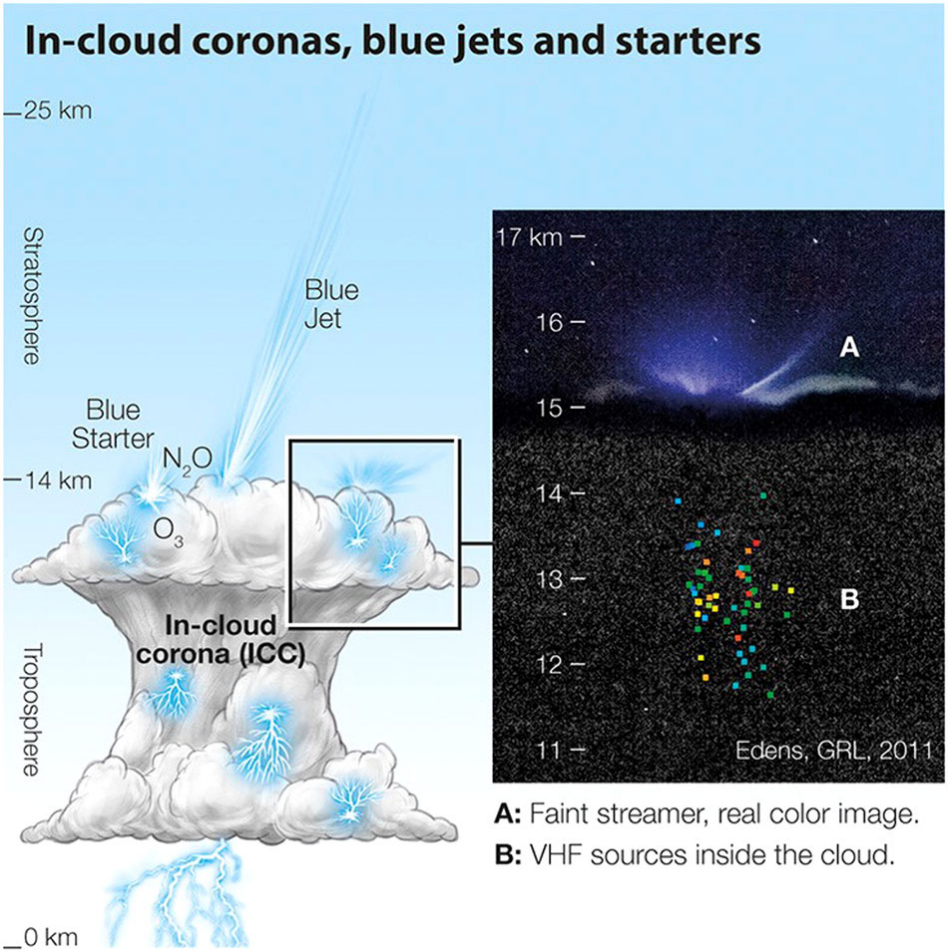
A schematic representation of corona discharges in thunderclouds^53^. The insert is a true colour photo of blue corona emissions, including a blue starter with the altitude of concurrent LMA sources shown as coloured dots increasing in time from blue to red^65^.

Lightning is one of the major sources of nitrogen oxides (NO_X_ = NO + NO_2_) throughout most of the free troposphere and the most important contributor in the upper troposphere, where the radiative forcing of ozone maximises. NO_X_ is the primary precursor for ozone, and the production efficiency in the UTLS region is 2–5 times higher than in the lower troposphere^68^. Whereas the effects of the lightning leader on the atmosphere have been discussed for several years, corona discharges represent a new pathway of chemical perturbations, which begs the question of their importance relative to lightning leaders. The ASIM studies have so far considered pure corona discharges (blue) and primarily in the upper levels of clouds where photons can escape the cloud in sufficient numbers to be detected by the instruments. Lightning flashes (red) are also commonly observed with intense blue emissions suggesting corona discharges are integral to the lightning processes, as expected (unpublished). Since thundercloud coronas occur in the vicinity of ordinary lightning or in isolation, they may represent a significant source of greenhouse gases. A schematic of corona discharges in a thundercloud is shown in Fig. 4.

In the channels of lightning strokes, neutral gas and electron temperatures reach up to ~40,000 K ~ 3.5 eV^70^. On the other hand, corona discharges in thunderclouds are formed by millions of streamer filaments^60,71,72^ that have non-uniform electric fields several times larger in the streamer head than in its body. The streamer head transmits energy to ambient electrons that become hotter (~8 eV) than in the lightning leader channels. The lightning leader is a hot, thermal plasma, whereas corona discharges have cold atoms, molecules and ions, with hot electrons that are not in thermal equilibrium. This leads to different efficiencies in the production of key chemical species. For example, laboratory studies show that ordinary lightning channels produce high amounts of nitric oxide (NO), while their direct ozone (O_3_) and nitrous oxide (N_2_O) production is negligible^73^. In contrast, coronas produce significant amounts of O_3_ and N_2_O, but only small amounts of NO^74–78^.

It is a priority to understand the sources and sinks of N_2_O because it is the third strongest greenhouse gas after CO_2_ and CH_4_ and can deplete ozone^79^. Since the early 1970s, reports have shown sudden, substantial ozone enhancements in or near thunderstorms that are thought to be produced in corona discharges;^80^ however, their global contribution is still highly uncertain. In addition to coronas, fertilised soils of agricultural areas are significant sources of N_2_O^81^, which can be transported to the UTLS region by deep convection^11^. Thus, current research aims to identify and quantify N_2_O sources and sinks and assess their importance.

We suggest that altitude-resolved spectral observations of coronas from the ISS and NBEs from the ground will allow the quantification of corona chemical activity. Spectral observations can also help unravel the underlying non-equilibrium kinetics of cloud corona streamers required for modelling perturbations to greenhouse gas agents. This will be a first step towards understanding the possible effects of coronas on the atmosphere’s radiation budget and the importance of coronas relative to lightning leaders.

### Altitude-resolved observations

Thunderstorm convection may transport and mix species directly produced by lightning and coronas with urban pollution and smoke from biomass burning to the UTLS region, affecting the radiation budget^11–13,82^. For example, the convective transport of SO_2_ might trigger the production of new ultrafine particles that changes the aerosol composition and radiation budget in the region^83,84^. And thunderclouds can enhance tropospheric O_3_ by wrapping and shedding stratospheric air^85^. The rapid spread of the anvil outflow in the horizontal direction may affect a large area (~100–1000 km in one direction) and its radiation balance^86^. Even a few days after the thunderstorm dissipated, this effect is measurable by airborne in-situ trace gas instrumentation^11^.

**Fig. 5:**
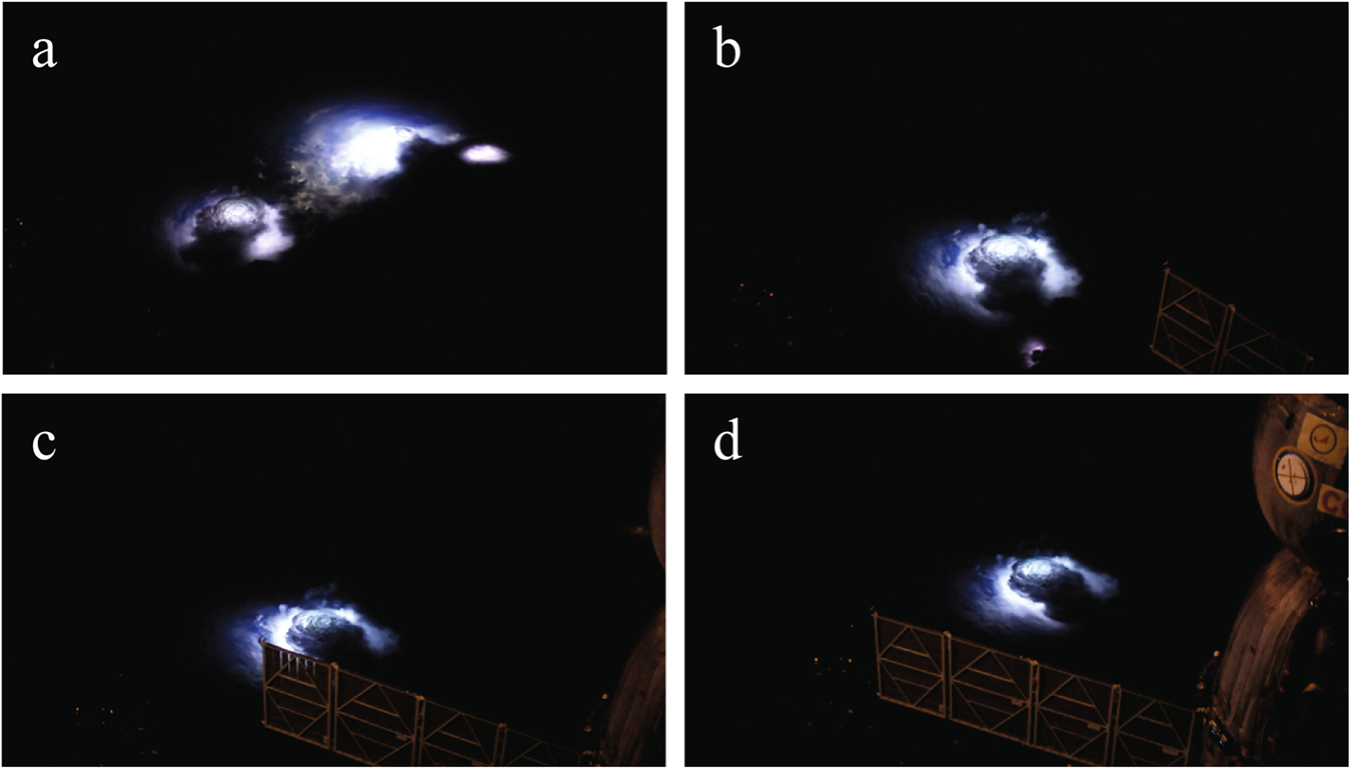
A time sequence of thunderstorm clouds viewed in a slanted perspective from the ISS as it passes over the storm. The sequence progresses clockwise from (**a**) to (**d**) (Credit NASA, ESA; Photo: Andreas Mogensen).

Clouds and aerosols are the sources of significant uncertainties in climate change predictions. In connection with thunderstorms, some outstanding questions relate, for instance, to the formation of deep convective events and to what extent they affect the moisture distributions within the UTLS region^87–92^. We suggest that these aspects can be addressed in slanted viewing geometry. The photos in Fig. 5 illustrate the potential of such measurements. At night, lightning inside the clouds give a spectacular view of the finer details of their structure and vertical extent. The smaller cloud turret is ~20–30 km across and reaches the tropopause at ~16 km altitude^54^. During the day, the structure can be detected from sunlight reflections. Slanted viewing from the ISS can then measure such phenomena as deep convective events and the formation of cloud turrets extending into the stratosphere, giving a full view of the clouds at a precision that exceeds that of the monitoring satellites in geostationary orbit.

Machine learning techniques applied to the lightning energy and spatial distribution measured by the GLM lead to an altitude within 1.5 km compared to measurements by an LMA^93^. Estimations of cloud top altitudes and location from stereo observations of lightning from GOES-R and -S, lead to ~5 km accuracy on the geographic location, ~240 m in mean altitude compared to the Advanced Baseline Imager (ABI) Cloud Height Algorithm (ACHA), with some kilometres spread in the estimation^94^. As with the stereo observations of GOES, a single instrument on the ISS can give position and altitude determinations comparable to the pixel resolution using the different perspectives of the clouds as the ISS passes over the storm. It requires that the storm cloud has recognisable structures observable in several instances during the overflight.

Estimates of chemical perturbations by lightning are based on, for instance, energy deposition in the atmosphere by strokes, observations from aircraft, balloons and the ground, laboratory experiments, and from different lightning parameterisations in chemistry-climate models^95^. Some models of lightning-generated NO_X_, like the NASA Lightning Nitrogen Oxides Model, can estimate the unmixed and otherwise environmentally unmodified vertical profile of lightning NO_X_, provided the altitude of lightning flashes and channel lengths are measured or assumed^96^. Such a model is well suited to ingest altitude-resolved activity. Whereas global climate models are not the best option to simulate thunderstorm impacts because their horizontal resolution leads to averages of regions that are large compared to the typical size of thunderstorms. Mesoscale models are better suited^97^.

### Opportunities with the research infrastructure of the coming years

**Fig. 6:**
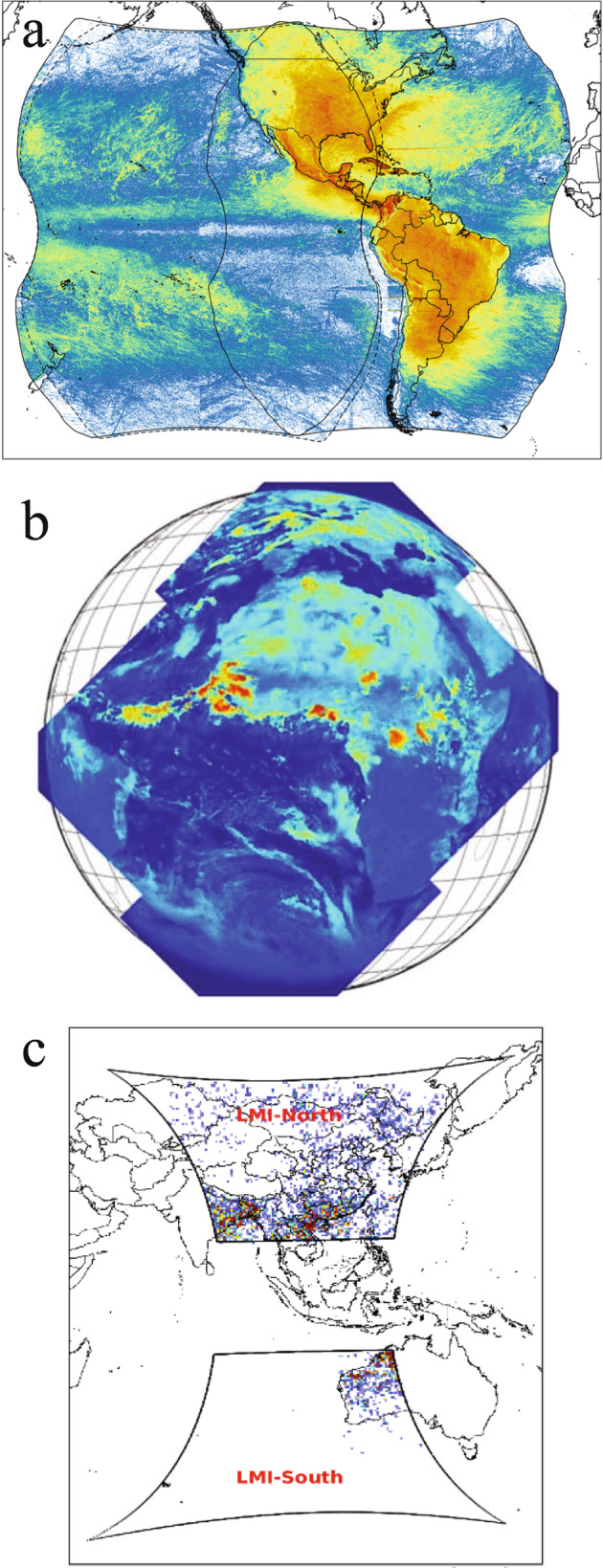
The coverage of geostationary lightning imagers. Flashes detected by the GLM on GOES-R and -S during 1 Dec 2018–31 May 2020 with the colour bar from 0,003 to 30 flashes/km^2^/month^103^ © American Meteorological Society. Used with permission (**a**), coverage of the four identical detectors of LI on MTG covering Africa and Europe^36^ © American Meteorological Society. Used with permission (**b**), and the LMI on FY4A with flashes per 0.5° × 0.5° bin/year from 0 to 1000. The instrument cover either one or the other hemisphere selected by satellite attitude changes^104^ (**c**).

With the launch of the LI on MTG-I, there will be almost global and continuous coverage of lightning from geostationary orbit with sensors based on the same principles as OTD/LIS. The range of GLM on GOES-R, S, LI on MTG and LMI on FY4A is shown in Fig. 6. Not shown is LMI on FY4B stationed at 105^O^E.

**Fig. 7:**
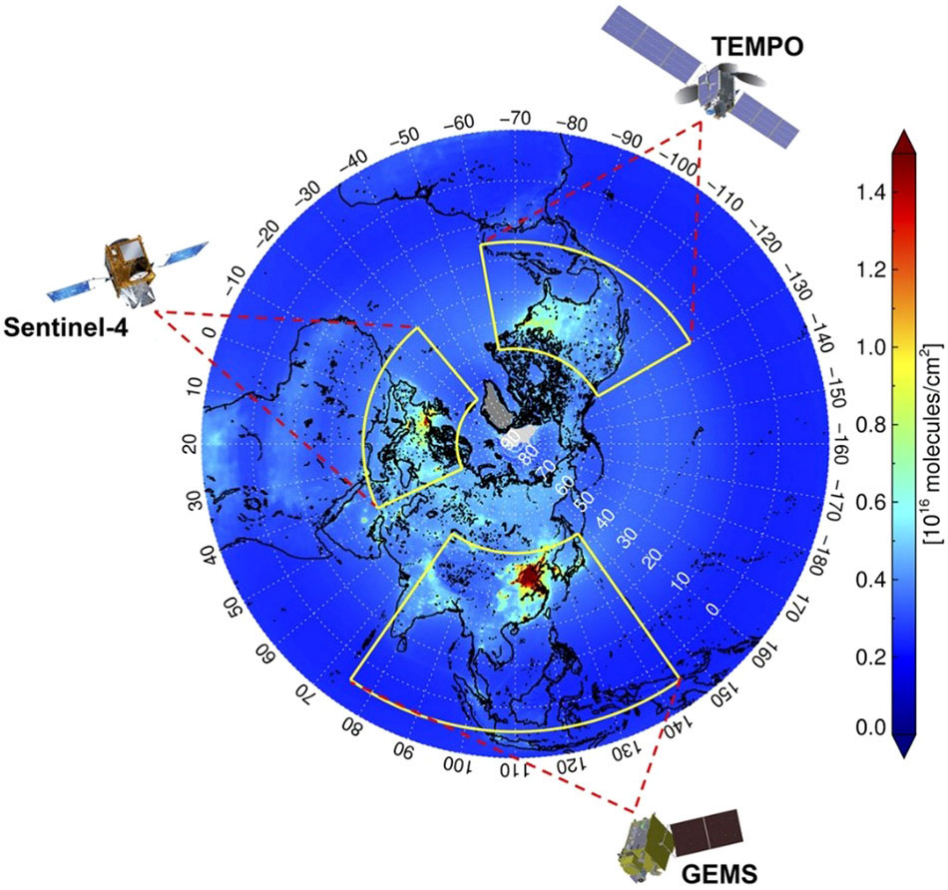
The Geostationary Air Quality Constellation covering the most polluted regions in the Northern Hemisphere. The background image is 10-yr average NO_2_ column densities observed by the Ozone Monitoring Instrument (OMI) on board the Aura satellite from 2005 to 2014, showing spatial coverage of GEMS over Asia, TEMPO over North America, and Sentinel-4 over Europe^98^ © American Meteorological Society. Used with permission.

Many trace gases will also be measured from geostationary orbit with the Air Quality Constellation. It consists of South Korea’s Geostationary Environment Monitoring Spectrometer on the GEO-KOMPSAT-2B satellite^98^, NASA’s Tropospheric Emissions: Monitoring of Pollution (TEMPO)^99^ on Intelsat 40e, expected to be launched in 2022, and ESAs Sentinel-4 instruments in 2023 to be carried by MTG-S^100^. Their coverage is shown in Fig. 7. The measurements will include, for instance, the total column densities of O_3_, NO_2_, SO_2_, HCHO, vertically resolved measurements of O_3_ and NO_2_, cloud optical thickness, fraction, altitude, aerosol column, optical thickness, type, layer height and absorbing index. Although aimed at detecting pollution in industrial regions of the Northern Hemisphere, the measurements also cover the major thunderstorm regions of the hemisphere and offer an opportunity to measure lightning perturbations to trace gas concentrations.

The upcoming launch of MTG-S with an Infra-red Sounder and an Ultraviolet Visible Near-infra-red spectrometer (Sentinel-4 instrument) will detect several trace species (O_3_, NO_2_, SO_2_, HCHO, CO, H_2_O, and aerosols)^100^. The MTG-I and -S offer an unprecedented opportunity to study the processes outlined above. The spatial and temporal resolution is 4 × 4 km^2^ and 30 min, which will allow for observations on the scale of a thunderstorm cell. Furthermore, the temporal evolution of O_3_ production from lightning NO_*X*_ might even be monitored in long-lived thunderstorms like Mesoscale Convective Systems.

Simultaneous observations by MTG and limb/slant pointing instruments on the ISS of lightning and corona activity, and other measurements with resolution in altitude, offers for the first time an opportunity to simultaneously monitor how thunderstorms and lightning can influence the chemical composition of the UTLS region. In addition to the temporal evolution of the column density of species, vertically resolved MTG-S measurements will be available for some of these species (SO_2_, H_2_O, formaldehyde (HCHO) and aerosols). They can be combined with the altitude-resolved measurements from the ISS, giving essential information for testing chemical models to study the climate effect of thunderstorms and lightning on the climate-sensitive UTLS region.

### Validation of measurements by geostationary lightning and trace species mappers

Planned and current space missions that observe thunderstorm clouds and electrical activity do so with nadir-pointing instruments. For instance, in the case of MTG, a slant-viewing instrument in LEO could be valuable for characterising the detection efficiency, the influence of cloud cover on radiance and geographic location accuracies. The highest flash rate storms, which are of significant interest for nowcasting, tend to have flashes with relatively weak optical emissions^101^. Capturing these weaker lightning discharges may prove essential. A slant-viewing instrument could assist in objectively characterising how MTG LI performs with respect to such flashes.

**Fig. 8:**
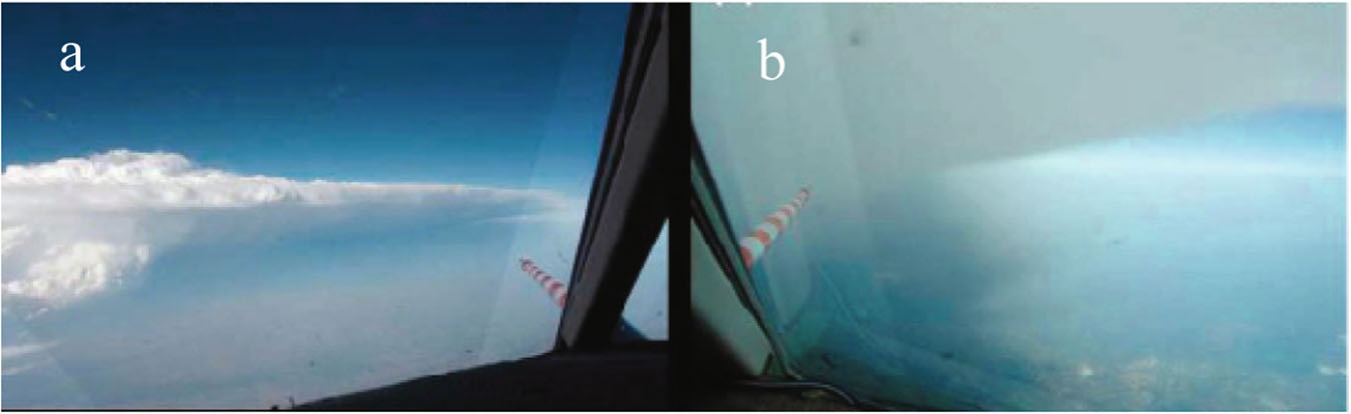
View from the cockpit of Falcon flight on 29–30 May 2012 over Oklahoma. On 29 May at 23:54 UTC (**a**) and 8 min later on 30 May at 00:02 UTC inside the lower boundary of the anvil outflow (**b**)^11^. (Photo R. Welser and A. Minikin, DLR).

Airborne, in-situ measurements in the vicinity of thunderstorms must provide ground truth of satellite measurements and models. Needed are measurements of trace species, estimates of specific trace species’ vertical column densities, and the vertical distribution of some species, such as O_3_ and NO_2_. Flights near deep convection are challenging and rare but have successfully been performed in the past in several studies around the world, as in the field experiment illustrated in Fig. 8. Combining measurements from aircraft with satellite observations of trace gases will help to interpret observations from either platform and allow more accurate comparisons between thunderstorm electrical activity and convection to the effects on the atmosphere’s composition.

### Outlook and Summary

The next decade promises to be a renaissance for studies of electric storms. The geostationary spacecraft constellations with instrumentation for detecting lightning, clouds and trace gas perturbations will allow long-duration monitoring of lightning activity and its effect on the atmosphere’s trace gas composition. We discuss how instruments in a slanted viewing geometry on spacecraft in low Earth orbit may resolve the altitude with a high spatial resolution that will help to quantify the occurrence and conditions for the generation of coronas and overshooting clouds. Improved models of the chemical perturbations by the electrical activity can be evaluated from satellite and simultaneous aircraft observations. Understanding the relationship between thunderstorm characteristics and atmospheric perturbations gained by instruments on the ISS or other low Earth orbit satellites can be applied to the global characterisation of thunderstorm activity by the geostationary lightning mappers to estimate the global effects. It will allow us to advance in the quest to understand how thunderstorms influence the atmosphere’s radiative properties and how climate change affects thunderstorm activity.

### Reporting summary

Further information on research design is available in the Nature Portfolio Reporting Summary linked to this article.

## Supplementary information

Reporting Summary Checklist
